# Central Auditory and Vestibular Dysfunction Are Key Features of Autism Spectrum Disorder

**DOI:** 10.3389/fnint.2021.743561

**Published:** 2021-09-29

**Authors:** Yusra Mansour, Alyson Burchell, Randy J. Kulesza

**Affiliations:** ^1^Department of Otolaryngology, Henry Ford Macomb Hospital, Detroit, MI, United States; ^2^Department of Anatomy, Lake Erie College of Osteopathic Medicine, Erie, PA, United States

**Keywords:** brainstem, hearing, vestibuar sytem, autism, screening tools

## Abstract

Autism spectrum disorder (ASD) is a neurodevelopmental disorder characterized by repetitive behaviors, poor social skills, and difficulties with communication. Beyond these core signs and symptoms, the majority of subjects with ASD have some degree of auditory and vestibular dysfunction. Dysfunction in these sensory modalities is significant as normal cognitive development depends on an accurate representation of our environment. The hearing difficulties in ASD range from deafness to hypersensitivity and subjects with ASD have abnormal sound-evoked brainstem reflexes and brainstem auditory evoked potentials. Vestibular dysfunction in ASD includes postural instability, gait dysfunction, and impaired gaze. Untreated vestibular dysfunction in children can lead to delayed milestones such as sitting and walking and poor motor coordination later in life. Histopathological studies have revealed that subjects with ASD have significantly fewer neurons in the auditory hindbrain and surviving neurons are smaller and dysmorphic. These findings are consistent with auditory dysfunction. Further, the cerebellum was one of the first brain structures implicated in ASD and studies have revealed loss of Purkinje cells and the presence of ectopic neurons. Together, these studies suggest that normal auditory and vestibular function play major roles in the development of language and social abilities, and dysfunction in these systems may contribute to the core symptoms of ASD. Further, auditory and vestibular dysfunction in children may be overlooked or attributed to other neurodevelopmental disorders. Herein we review the literature on auditory and vestibular dysfunction in ASD. Based on these results we developed a brainstem model of central auditory and vestibular dysfunction in ASD and propose that simple, non-invasive but quantitative testing of hearing and vestibular function be added to newborn screening protocols.

## Introduction

### Autism Spectrum Disorder

Autism spectrum disorder (ASD) is a developmental disability associated with impairment in social, communicative, and behavioral domains (CDC.gov, 2021). ASD affects approximately one in 54 children and is four times more common in males. While difficulties with hearing and balance are not diagnostic signs or symptoms, children or adults with a diagnosis of ASD may have difficulty hearing or attending to speech or vocalizations despite being able to hear other environmental sounds and they may have abnormal responses to sounds. Further, large scale studies suggest that most if not all individuals with ASD have some degree of auditory dysfunction (Greenspan and Wieder, 1997) and several studies indicate brainstem and cerebellar pathological changes in ASD (Ornitz, 1969; Bauman and Kemper, 1985; Courchesne et al., 1987, 1988, 1994a,b; Ogawa, 1989; Scott et al., 2009). Herein, we review auditory and vestibular dysfunction in ASD and propose the incorporation of these modalities into screening for ASD.

### The Auditory System

**Figure 1:**
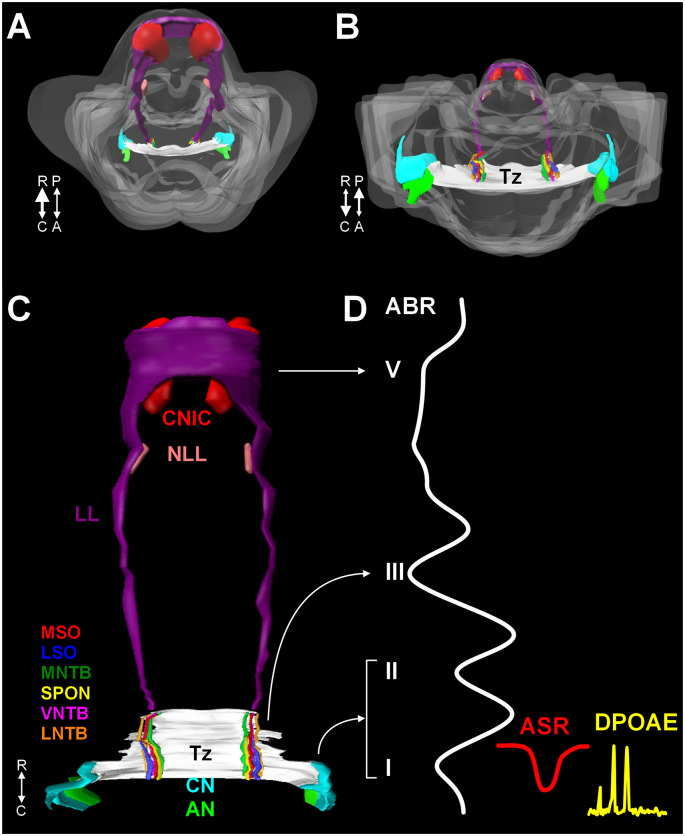
A 3D reconstruction of the human auditory brainstem. Images **(A)** through **(C)** show 3D volume renderings of nuclei and tracts of the human auditory brainstem. Image **(A)** shows a rostral to caudal view (from midbrain down to the medulla) and **(B)** shows a caudal to rostral view (from medulla up to midbrain). Image **(C)** shows a posterior view (viewed from posterior to anterior). In **(A)** and **(B)**, the contour of the brainstem is indicated in gray. A key to the colors and nuclei/tracts is shown in figure **(C)**. The CN and SOC nuclei are limited to the rostral medulla and caudal pons. The LL extends from the caudal pons to the CNIC. Figure **(D)** shows examples of ABR, ASR, and distortion product DPOAE recordings juxtaposed to the levels of the auditory pathway they measure. Numbers in roman numerals indicate specific waves of the ABR. ABR waves I and II correspond to the AN and CN, wave III corresponds to the SOC. Waves IV and V correspond to the LL and CNIC respectively. Abbreviations: R, rostral; C, caudal; P, posterior; A, anterior; DPOAE, distortion product otoacoustic emission.

The mammalian auditory system begins with bilaterally situated external ears or pinnae, that serve to collect and funnel sound pressure waves through the external auditory meatus towards the tympanic membrane. Vibrations of the tympanic membrane are transferred through the ossicles in the middle ear (tympanic cavity) to the oval window. Mechanical vibrations at the oval window transduce this energy to fluid waves of endolymph in the cochlear duct. These fluid waves activate mechanoreceptive inner hair cells in the organ of Corti in the cochlea. The central auditory pathway originates with bipolar neurons in the spiral ganglion. These neurons collect information from inner hair cells through their peripheral processes and relay this information via central axons to both the dorsal and ventral cochlear nuclei (DCN and VCN, respectively) in the rostral medulla (Figures 1A–C). Neurons in the VCN project bilaterally to the superior olivary complex (SOC; in the caudal pons) through the trapezoid body (Tz) and the contralateral inferior colliculus (IC; midbrain) through the lateral lemniscus (LL; Figures 1A–C). The SOC is a collection of brainstem nuclei, and each nucleus contributes a unique circuit subserving a specific function. As a group, the SOC plays prominent roles in localization of sound sources, coding temporal and spectral features of sound, and descending modulation of the organ of Corti. Along the LL, there are ventral, intermediate, and dorsal nuclei of the lateral lemniscus (VNLL, INLL, and DNLL, respectively) that receive input from the VCN and SOC and project to the inferior colliculus (IC). The IC includes a central nucleus (CNIC), a dorsal cortex, and an external cortex. The CNIC forms an essential component of the ascending auditory pathway and sends a major projection to the medial geniculate in the thalamus and specifically the ventral nucleus of the medial geniculate (vMG). The vMG projects through the internal capsule to the primary auditory cortex (A1).

Humans have a comparatively narrow range of hearing sensitivity but are excellent low-frequency listeners. The SOC is subject to sometimes drastic interspecies variation but the core nuclei are modified to meet the specific hearing needs of the animal; accordingly, the human SOC is specialized for encoding and localizing lower frequency sounds and includes a prominent medial superior olive (MSO; Kulesza, 2007). The human MSO is composed of a thin column of neurons and each neuron forms both a medial and lateral dendrite (Kulesza, 2007; Mansour and Kulesza, 2021). Human MSO dendrites are symmetric and are distributed into the peri-MSO fields (Mansour and Kulesza, 2020, 2021). These dendrites serve to collect information from both ears: the lateral dendrite receives input from the ipsilateral ear and the medial dendrite receives input from the contralateral ear. Neurons of the MSO are often referred to as coincidence detector neurons since they function to encode differences in arrival time of sounds between the two ears—this is known as the interaural time difference (ITD). Therefore, the normal number and morphology of MSO neurons and their dendrites are required for normal ITD coding.

Along with the ascending auditory pathway described above, there is a descending pathway that begins in the cerebral cortex that includes neurons at each level of the pathway, and terminates in the cochlea (see Schofield, 2010 for a detailed review). This descending circuit is complex and integrates auditory and non-auditory inputs. The final neurons in this descending pathway are situated in the SOC and comprise two unique circuits: a medial olivocochlear system (MOC) and a lateral olivocochlear system (LOC). Neurons compromising the MOC reside mainly in the ventral nucleus of the trapezoid body (VNTB) and these neurons project to outer hair cells in the organ of Corti. This projection results in the contraction of outer hair cells, serving to reduce cochlear output to filter out background noises when listening in noisy environments. Neurons of the LOC are situated in and around the lateral superior olive (LSO). LOC neurons send axons to the ipsilateral cochlea and innervate auditory nerve axons that innervate inner hair cells. Together, olivocochlear neurons modulate the function of the cochlea to protect the cochlea from damage by loud sounds and for selective listening in background noise.

### The Vestibular System

**Figure 2:**
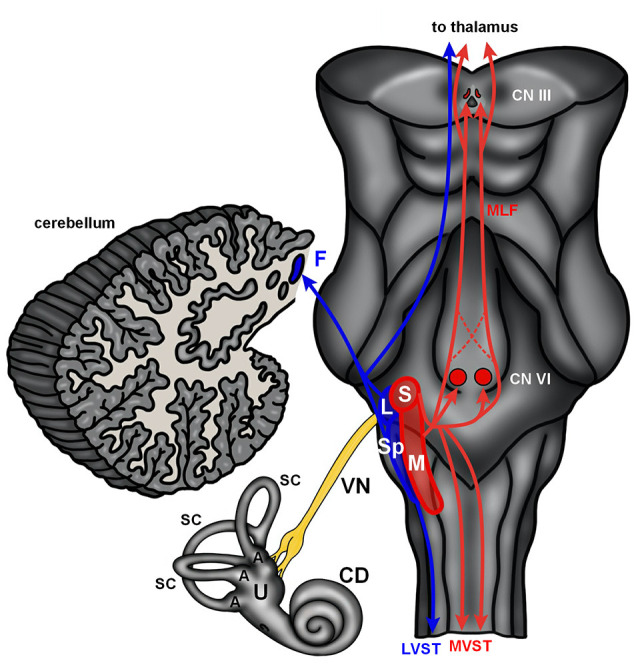
The human vestibular brainstem. A schematic of the human vestibular pathway and vestibulocerebellum is shown. Vestibular hair cells are housed in cristae found in the ampulla (A) of the three semicircular canals (SC), and macula situated in both the utricle (U) and saccule. These hair cell collections are innervated by the vestibular division of the vestibulocochlear nerve. The vestibular nerve (VN) projects to the vestibular nuclei (red and blue contours) that extend from the medulla into the caudal pons. The lateral (L) and spinal (Sp) vestibular nuclei project to the fastigial nucleus (F) in the cerebellum and spinal cord *via* the lateral vestibulospinal tract (LVST). The superior (S) and medial (M) nucleus project to the spinal cord *via* the medial vestibular spinal tract (MVST) and nuclei controlling extraocular muscles (CN VI—abducens nucleus; CN III—oculomotor nucleus) *via* the medial longitudinal fasciculus (MLF). The vestibular nuclei also send projections to the thalamus. Abbreviations: CD, cochlear duct.

The mammalian vestibular system begins with delicate, endolymph-filled membranous labyrinths encased in each temporal bone (Lysakowski and Goldeberg, 2004). Each membranous labyrinth includes a cochlear duct, two enlarged sac-like structures—the utricle and saccule, and three semicircular canals (Figure 2). The utricle and saccule include collections of mechanoreceptive hair cells arranged in maculae (Lysakowski and Goldeberg, 2004). The stereocilia of the macular hair cells are embedded in an otolith membrane. Movements of the head (e.g., up and down) cause inertial movements of the otolith and mechanical activation of macular hair cells. This serves to encode linear motion and orientation of the head relative to gravity. The base of each semicircular canal includes an ampulla where it joins the utricle and each ampulla contains a crista ampullaris. The cristae are composed of mechanoreceptive hair cells with stereocilia embedded in a gelatinous matrix known as the cupula. Movements of the cupula coincide with rotational movements of the head. Vestibular hair cells are innervated by peripheral processes of bipolar neurons located in the vestibular (Scarpa’s) ganglion. Neurons in the vestibular ganglion send central projections to the cerebellum and four vestibular nuclei residing along the floor of the fourth ventricle in the medulla and pons (Figure 2). The superior and medial vestibular nuclei (Figure 2, red) receive their major inputs from the cristae and project along the medial vestibulospinal tracts and medial longitudinal fasciculus to nuclei controlling the extraocular muscles and to the cervical spinal cord to control gaze and maintain a stable platform for the eyes. The lateral vestibular nucleus (Deiters’ nucleus) receives input from the cristae and macula and projects through the lateral vestibulospinal tract to influence postural reflexes (Figure 2, blue). The spinal (descending) nucleus receives input from the otolith organs and projects to the cerebellum, reticular formation, and spinal cord to regulate posture. Information from the vestibular ganglion and vestibular nuclei target the fastigial nucleus and flocculonodular lobe of the cerebellum (Figure 2, blue). Together, the vestibular apparatus and associated central connections encode movements of the head and direct adjustments to eye position, muscle tone, and body posture (Lysakowski and Goldeberg, 2004). Like the auditory hair cells, the vestibular sensory organs receive efferent innervation from cholinergic neurons near the genu of the facial nerve (Warr, 1975).

### Autism Spectrum Disorder and CN VIII Dysfunction

#### Auditory Dysfunction in ASD

Autism spectrum disorder (ASD) is a neurodevelopmental disorder characterized by difficulties in social, communicative, and behavioral domains (CDC.gov, 2021). The most recent reports indicate that ASD impacts one in 54 children with a strong predilection for males (CDC.gov, 2021). One of the key signs/symptoms of ASD is abnormal responses to sensory stimuli. This can manifest as hypersensitivity or overreactions involving somatic sensations (touch) or special senses (smell, vision, and hearing). Beyond abnormal responses to sound, auditory dysfunction is present in most, if not all individuals with ASD (Greenspan and Wieder, 1997; Tomchek and Dunn, 2007; Bolton et al., 2012). This dysfunction ranges from deafness to hyperacusis and often includes difficulty listening to background noise and understanding speech (Roper et al., 2003; Alcántara et al., 2004; Khalfa et al., 2004; Szelag et al., 2004; Teder-Sälejärvi et al., 2005; Gravel et al., 2006; Tharpe et al., 2006; Russo et al., 2009). Collet et al. (1993) were the first to suggest the hyperacusis observed in ASD might be related to abnormalities in the MOC system. Consistent with these reports, hearing difficulties have been proposed as a cardinal indicator of ASD (Osterling and Dawson, 1994).

Interestingly, involvement of the auditory system in ASD was suspected in the original report of autistic children in 1943. One of the common findings in these children was difficulty with language and hypersensitivity to loud noises (Kanner, 1943). Observations from a study of auditory-evoked potentials further supported the idea of auditory involvement (Ornitz et al., 1968). Additional studies of individuals with ASD implicated problems with processing language (Hermelin and Frith, 1971), focusing on multiple sounds or stimuli (Reynolds et al., 1974), conductive hearing loss (Smith et al., 1988) and hyposensitivity, or hypersensitivity depending on the stimulus modality (Ornitz et al., 1968; Hayes and Gordon, 1977; Rosenhall et al., 1999). A number of studies also suggested difficulties in processing complex sounds such as speech. Specifically, (Koegel and Schreibman, 1976) reported on a child with ASD who appeared to be deaf for complex sounds (white noise) but responded to environmental sounds at normal thresholds. A review of 1st birthday home videos revealed that children with ASD often failed to orient to their name being called (Osterling and Dawson, 1994), have a clear preference for noise or non-verbal sounds over speech and vocalizations (Klin, 1991, 1993) or frank impairment for speech sounds (Ceponiene et al., 2003; Jeste and Nelson, 2009; Bidet-Caulet et al., 2017; Jayanath and Ozonoff, 2020).

The auditory dysfunction in ASD has been studied and characterized by numerous researchers using fairly simple, non-invasive techniques. The acoustic stapedial reflex (ASR) has been utilized to examine the function of the lower auditory brainstem, facial nucleus, and contraction of the stapedius muscle in subjects with ASD with some conflicting results (Figure 1D; Gravel et al., 2006; Tharpe et al., 2006; Gomes et al., 2008). Specifically, only Gomes found that subjects with ASD had lower thresholds relative to neurotypical (NT) individuals. (Gravel et al., 2006) found no significant differences between control children and subjects with ASD in cohorts that were matched by age and hearing threshold. We examined the ASR in a group of 29 neurotypical children (ages 7–17 years) and 54 children and young adults with high-functioning ASD (ages 4–23 years; Lukose et al., 2013). The subjects with ASD had significantly lower thresholds (i.e., hypersensitivity), and significantly longer response latencies. The longer latencies were most commonly observed in response to a 1 kHz tone presented in the ipsilateral ear. In all NT subjects, ipsilateral reflex responses always occurred at shorter latencies compared to contralateral reflex responses regardless of tone frequency. However, in subjects with ASD, this clear and predictable pattern of slower ipsilateral responses was not always found. Specifically, when we stimulated the left ear of subjects with ASD, the ipsilateral reflex responses occurred at a significantly longer latency compared to the contralateral response. Finally, in our study of the ASR, 97% of subjects with ASD had at least one response outside the 95% confidence interval of NT responses. Regardless, our results are based on a relatively small group of children and young adults—whether these findings can be generally extended to subjects with ASD will require further investigation.

The ABR is a sound-evoked response of synchronized brain activity and each peak or wave corresponds to a particular level of the auditory brainstem pathway (Figure 1D). The ABR has provided the most insight into the function of brainstem centers in ASD. The majority of studies of the ABR in subjects with ASD over the past 40 years provide evidence that subjects with ASD have smaller amplitudes in waves I, II, III, IV, and V (Ornitz et al., 1972; Gillberg et al., 1983; Martineau et al., 1987, 1992; Klin, 1993), longer latencies between waves I-III and waves I-V (Taylor et al., 1982), and longer latencies/slower responses (Ornitz, 1969; Student and Sohmer, 1978; Rosenblum et al., 1980; Sohmer, 1982; Tanguay et al., 1982; Gillberg et al., 1983; Sersen et al., 1990; Thivierge et al., 1990; Wong and Wong, 1991; Maziade et al., 2000; Kwon et al., 2007; Roth et al., 2012; Azouz et al., 2014; Taş et al., 2017; Miron et al., 2018, 2021; Ramezani et al., 2019; Delgado et al., 2021; reviewed in Talge et al., 2018). These longer latency and lower amplitude responses have been attributed to the immaturity of brainstem circuits (Li et al., 2020). A recent study showed delays in speech-based ABRs (Chen et al., 2019) and reduced binaural interaction components (BIC) of the ABR in subjects with ASD (ElMoazen et al., 2020). The latter study also found a significant positive correlation between the amplitude of the BIC ABR waveform and both language and social scores in subjects with ASD (ElMoazen et al., 2020).

The literature provides convincing evidence that ABRs can be used as a screening instrument for the risk of ASD and/or other neurodevelopmental disorders. Specifically, a prospective study of ABRs found that young children (birth to 3 months) with longer wave V latencies and I-V interpeak latencies were later diagnosed with ASD (Miron et al., 2016). Consistent with observed asymmetries in ASR and otoacoustic emissions (OAEs; see below), these authors found longer III-V interpeak latencies when stimulating the right ear only. In fact, these changes in wave V have a positive predictive value of 78% and a negative predictive value of 73% for wave V. These differences in subjects with ASD can be further illustrated with masking experiments. Such paradigms revealed abnormal interpeak latencies between waves I-V and III-V (Thivierge et al., 1990), the reduced amplitude of wave III (Källstrand et al., 2010), and asymmetric masking by contralateral noise (Khalfa et al., 2001).

While ASR and ABRs examine brainstem circuits, the function of the cochlea can be evaluated using otoacoustic emissions (OAEs). Similar to the asymmetry seen in ASR testing, subjects with ASD exhibited abnormal OAEs with marked asymmetry (Khalfa et al., 2001) and significantly reduced responses in the 1 kHz ranges (Bennetto et al., 2017). However, other researchers have found hypersensitivity or elevated responses (Danesh and Kaf, 2012; Taş et al., 2017). These conflicting results might be attributed to differences in subjects, equipment, and/or interpretation of the data. Also, the direct involvement of the cochlea in ASD is unclear. While OAEs provide an objective measure of the auditory function, its value as a screening tool is unclear. Beyond differences in ASR, OAEs, and ABRs, there is abundant evidence for additional problems in auditory processing in ASD. Specifically, subjects with ASD have difficulties with temporal processing (Russo et al., 2008; Bhatara et al., 2013; Foss-Feig et al., 2017), difficulties listening in the presence of background noise (Alcántara et al., 2004; Teder-Sälejärvi et al., 2005) and problems with sound localization tasks (Osterling and Dawson, 1994; Baranek, 1999; Werner et al., 2000; Dawson et al., 2004; Lodhia et al., 2014, 2018). Finally, there are reports of dysfunction in the auditory forebrain. This includes weaker interhemispheric projections, stronger projections from the thalamus to the cortex in subjects with ASD (Linke et al., 2018), and right-left asymmetries of the secondary auditory cortex (Orekhova et al., 2012; Azouz et al., 2014). Additionally, there is evidence for abnormalities in cortical evoked auditory potentials (reviewed in O’Connor, 2012). It is unclear if these forebrain abnormalities result from dysfunction of brainstem centers or if the auditory forebrain is an additional primary site of injury in ASD. Therefore, it seems very likely that the difficulties children with ASD have developing language is intimately related to problems encoding and understanding the complex temporal and spectral features of speech.

Taken together, these studies implicate the lower auditory brainstem as a key site, if not the origin of abnormal circuitry and dysfunction in ASD. These changes can be identified in young children and can be assessed at birth. It is important to recognize that identification of these differences requires careful analysis of the responses. Newborn hearing screenings are often superficial and many children with auditory dysfunction pass these newborn screens or are missed on follow-up testing. In fact, a recent study demonstrates that childred who failed newborn ABR screens but were diagnosed with normal hearing at follow-up were five to 10 times more likely to be diagnosed with ASD (Tu et al., 2020). We hypothesize the majority of children that will be diagnosed with ASD have these hearing issues at birth and are missed by routine newborn screening because they have clinically normal hearing thresholds. Consistent with this hypothesis, only a small number of children with ASD have abnormal pure tone audiometry results, but when combined with comprehensive auditory screening (including audiometry, tympanometry, acoustic reflexes, OAE, and ABR) more than half of the subjects have an abnormal result; regardless subjects with ASD who pass these screens can still have difficulty with speech and language tasks (Demopoulos and Lewine, 2016).

#### Auditory Brainstem Dysmorphology in ASD

A number of imaging studies revealed smaller cerebella and brainstems in subjects with ASD (Courchesne et al., 1988, 1994a,b; Gaffney et al., 1988; Murakami et al., 1989; Ciesielski et al., 1990; Hashimoto et al., 1992, 1993, 1995; Kleiman et al., 1992; Piven et al., 1992). Postmortem neuropathological studies confirmed that subjects with ASD have consistent dysmorphology in the brainstem and cerebellum (Williams et al., 1980; Bauman and Kemper, 1985; Bauman, 1991; Ritvo et al., 1986; Arin et al., 1991; Rodier et al., 1996; Bailey et al., 1998; Kulesza and Mangunay, 2008; Kulesza et al., 2011; Lukose et al., 2015; Mansour and Kulesza, 2020). Specifically, these studies revealed fewer cerebellar Purkinje cells and hypoplasia of the facial nucleus and SOC. Further investigations by Wegiel and coworkers revealed multifocal heterotopias and dysplasias in the forebrain and cerebellum and significantly fewer cerebellar Purkinje cells (Wegiel et al., 2010, 2014). Together, these findings support multiple sites of impaired neurogenesis, neuronal migration, and/or neuron survival in ASD (Wegiel et al., 2010, 2014).

Some of the first evidence for focal brainstem deficits in ASD was the work of Rodier and colleagues (Rodier et al., 1996). These researchers found significant hypoplasia of the facial nucleus and SOC, abnormal bundles of axons related to the hypoglossal nucleus, and significant reduction in the rostrocaudal length of the pons. Based on their finding of changes in the SOC, together with reports of gross brainstem dysmorphology and hearing difficulties in ASD, we hypothesized these hearing difficulties are directly related to dysmorphology of lower auditory brainstem centers. We investigated this hypothesis first by studying the brainstems of five subjects with ASD (8–32 years of age) and two typical developing controls (26–29 years of age; Kulesza and Mangunay, 2008). A preliminary study of these specimens revealed noticeable changes in the MSO and we, therefore, focused our analysis on this nucleus. Our previous study of over 85 brainstems from NT subjects revealed the human MSO is composed of a thin column of 13–14,000 neurons (Kulesza, 2007; Lukose et al., 2015; Mansour and Kulesza, 2021) each emitting medial and lateral dendrites collecting input from the contralateral and ipsilateral ears, respectively (Mansour and Kulesza, 2021). The structure and arrangement of MSO neurons is essential to their function of encoding ITDs. We discovered that the MSO from subjects with ASD have significantly smaller neurons and the majority of these neurons have round/oval soma. We also found these neurons are abnormally oriented within the MSO. We then studied a larger cohort of brainstems, from nine subjects with ASD (2–36 years of age) and four NT individuals (4–32 years of age; Kulesza et al., 2011). In this study we analyzed all six SOC nuclei and found fewer and significantly smaller neurons in five of the six constituent nuclei; only the VNTB was unaffected (Lukose et al., 2011). Subjects with ASD also had extracellular eosinophilic fibers, hypergliosis around the MSO, and in two subjects (two of nine; ~22%) clusters of ectopic neurons in the pontine tegmentum. We then extended this to an even larger cohort including 10 NT subjects (3–32 years of age), 16 subjects with ASD (5–56 years of age) and 12 subjects with ASD and a duplication of the *q* region of chromosome 15 [dup(15q)] (5–39 years of age; Lukose et al., 2015). In this cohort, we found fewer and smaller neurons in all SOC nuclei except the VNTB. Consistent with our previous studies, the MSO was the most severely affected nucleus in the SOC. In NT subjects, the MSO included about 13,000 neurons. In subjects with ASD or ASD + dup(15q), there were only about 5,400 neurons in the MSO and these neurons were significantly smaller, more round, and abnormally arranged in the nucleus. The peri-MSO was also significantly smaller in subjects with ASD (Mansour and Kulesza, 2020) and we interpret this finding to suggest that dendrites of MSO neurons are significantly shorter and less complex than NT subjects. Consistent with this hypothesis, human MSO neurons from subjects with ASD are smaller, more circular, and emit smaller caliber primary dendrites (Kulesza et al., 2011; Lukose et al., 2015). In this cohort, we compared the total number of MSO neurons with the subject’s Autism Diagnostic Interview-Revised (combined social, communication, and behaviors scores); however, there was no relationship between these values. Our previous studies of the human MSO from NT subjects revealed round/oval neurons are more common in younger subjects (<10 years of age). Therefore, we interpret the presence of round/oval neurons in the adult MSO of subjects with ASD to indicate brainstem immaturity or arrested development. In this study, we also found ectopic clusters of neurons in the caudal pontine tegmentum. We believe these ectopic cells to be neurons lost during migration—neurons possibly destined for the SOC that never arrived and therefore fail to participate in auditory circuits. Recently, we constructed 3D volumetric models of the human SOC nuclei from young neurotypical subjects and subjects with ASD (Mansour and Kulesza, 2020). Consistent with our previous studies, we found that in ASD, all of the SOC nuclei except the VNTB occupy smaller volumes and this was not related to overall brain size. Our findings of severe dysmorphology and neuronal loss in the MSO of subjects as young as 2 years old and the observation of Purkinje cell loss with an intact inferior olive are consistent with developmental dysfunction before 28–30 weeks of gestation (Bauman and Kemper, 2005). Further, we propose that deficits in higher degree auditory function result from abnormal coding of temporal and spectral features by the cochlear nuclei (CN) and SOC. Based on our consistent observations of dysmorphology, we propose the MSO be added to the claustrum and cerebellar Purkinje cells as neuropathological hallmarks of ASD (Wegiel et al., 2014).

#### Modified Auditory Brainstem Circuitry in ASD

It is important to note that ASD is a spectrum disorder and not all individuals are affected to the same degree. Indeed, our morphological studies show not all subjects have the same degree of hypoplasia, the number of ectopic neurons, or gliosis. We attribute hyposensitivity to sound to result from fewer neurons, smaller axons, and abnormal ascending projections to the CNIC. These changes undoubtedly contribute to changes in the ABR and ASR, problems with vocalizations and localization of sound sources and likely contribute extensively to dysfunction of the auditory forebrain. We believe that hypersensitivity and difficulty listening in background noise likely result from changes in the number of SOC or facial nucleus neurons, and/or their connectivity or alterations in the organ of Corti.

**Figure 3:**
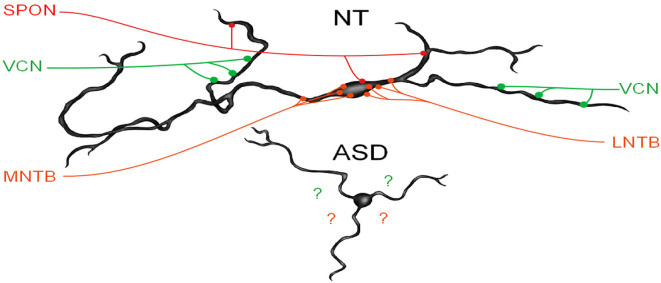
MSO dysmorphology in ASD. The top image shows a reconstruction of a human MSO neuron (Mansour and Kulesza, 2021). Human MSO neurons have slender cell bodies that form symmetric dendrites on both the medial and lateral sides of the MSO cell column. MSO neurons receive symmetric glutamatergic input from both ipsilateral and contralateral VCN and these are distributed symmetrically on distal dendrites, glycinergic inputs are distributed primarily on the cell body and proximal dendrites and originate from the medial and lateral nuclei of the trapezoid body (MNTB and LNTB, respectively). Additionally, there are GABAergic inputs from the superior paraolivary nucleus (SPON). MSO neurons integrate these precisely arranged and timed inputs to extract spectral and temporal features of sound. MSO neurons in subjects with ASD lack the same morphology and distribution and the arrangement of these inputs are unknown.

We have recently demonstrated that human MSO neurons form symmetric medial and lateral dendrites, that glycinergic inputs are segregated to the cell body and proximal dendrites while excitatory inputs are arranged further distally (Mansour and Kulesza, 2021). These features are crucial for normal MSO function. In subjects with ASD, not only are there fewer MSO neurons but these neurons are smaller, their dendrites are significantly shorter and issued in almost random directions (Figure 3). We believe that in ASD, the reduction in the size of dendrites results in less area for collecting and integrating inputs from the ipsilateral and contralateral ears, and the random arrangement likely results in a poorly organized tonotopic map in the MSO (Figure 3). As a result, MSO neurons are not able to precisely extract timing and spectral information from their binaural inputs. Furthermore, there is a significant reduction in the number of MSO neurons and likely reduced projection from the MSO and SOC. Together, these findings suggest that not only is there a significant reduction in the MSO and SOC projection to the CNIC, but also this input is not carrying the same type/quality of information about the auditory environment. We proposed that the changes observed by many researchers in ABRs can be attributed to the reduced number of brainstem neurons, smaller, poorly myelinated axons, and/or abnormal patterns of activation in the auditory nerve (AN), Tz, LL. Accordingly, we believe that subjects with ASD fail to encode many complex features of vocalizations and may miss subtle features and auditory cues.

The VNTB functions in multiple aspects of hearing including descending modulation of the cochlea and fine-tuning local and ascending circuits. In subjects with ASD, we have found no changes in size or number of VNTB neurons or volume occupied by this nucleus. The descending projection from the VNTB to the cochlea has an identified role in filtering sound necessary for listening in background noise. However, subjects with ASD have difficulties listening to background noise. We have yet to assess the connectivity of the VNTB in human subjects. But we have examined projections of the VNTB in an animal model of ASD and found no differences from control animals (this issue, Mansour and Kulesza, 2021). However, in human subjects, it is unclear if VNTB projections are intact and/or have normal function. A recent study showed abnormal OAEs in subjects with ASD (Bennetto et al., 2017) implicating dysfunction of the organ of Corti. Morphological studies of the cochlea, including afferent and efferent innervation, from subjects with ASD, will clarify the structural and functional roles of the sensory receptors in this condition.

#### Vestibular and Cerebellar Issues in ASD

A number of early morphological studies of subjects with ASD suggested the brainstem as the origin of dysfunction (Ornitz, 1969; Ogawa, 1989). Some of the first support for this hypothesis was provided by imaging studies revealing changes in the cerebellum (Bauman and Kemper, 1985; Courchesne et al., 1987, 1988, 1994a,b; Scott et al., 2009). Specifically, these studies found hypoplasia of the cerebellar vermis involving lobules VI and VII and more widespread involvement of the vermis including lobules I-V and VIII-X (Courchesne et al., 1994a,b; Levitt et al., 1999). Consistent with these imaging studies, microscopic studies of the cerebellum revealed variable changes in the size of cerebellar Purkinje cells, with fewer Purkinje cells in the posterior cerebellum and occasionally the vermis and fewer neurons in the deep cerebellar nuclei in ASD (Bauman et al., 1995; Bauman, 1996). However, other studies have shown no difference in cerebellum size/volume between subjects with ASD and NT subjects (Scott et al., 2009). These conflicting results in cerebellum morphology might be attributed to variations amongst study subjects, ASD severity, or diagnoses. Indeed, detailed and systematic studies of the cerebellum and brainstem are needed to fully appreciate how these brain regions are impacted in ASD.

While motor signs and symptoms are not diagnostic features of ASD, deficits in motor skills and coordination are receiving more attention (Gernsbacher et al., 2008; Bhat et al., 2012; Lai et al., 2014; Sacrey et al., 2014). Further, several studies have demonstrated dysfunction of the vestibular system and/or vestibular circuits in the cerebellum. A number of early studies indicated that subjects with ASD have postural issues, problems with balance (Ritvo et al., 1969; Ornitz, 1970; Molloy et al., 2003; Smoot Reinert et al., 2015), and abnormal responses to vestibular stimulation (Slavik et al., 1984). Specifically, after rotational stimulation subjects with ASD had a slower onset of the primary nystagmus response and fewer beats during the secondary response (Ornitz et al., 1985). Subjects with ASD have irregular patterns of horizontal gaze, abnormal rotation-induced vestibulo-ocular reflexes (VOR), and VOR-based tasks that were attributed to abnormal cerebellum and brainstem circuitry (Carson et al., 2017; Caldani et al., 2020). VOR has been proposed to serve some diagnostic value in ASD (Thabet, 2014). Young subjects with ASD have longer latency saccades compared to age-matched neurotypical controls (Furman et al., 2015). In a recent population-based study of over 10 million neurotypical and 61,000 children with ASD, it was found that ophthalmic dysfunction was far more common in children with ASD (Chang et al., 2021). Specifically, ophthalmic dysfunction was present in ~14% of children with ASD, that strabismus was four times more common, and nystagmus was nearly 10 times more common in ASD. Interestingly, in a study of 62 subjects with ASD, end-stage nystagmus was associated with better performance on language, cognitive and motor screens (Pineda et al., 2015). Vestibular involvement in ASD is not as clear as auditory issues—in a study of 79 subjects with ASD ranging in age from 5–52 years of age there was no difference in responses to rotational stimulation (Furman et al., 2015) and in a study of 13 children with ASD there was no difference in post-rotary nystagmus (Goldberg et al., 2000). But, children with ASD have abnormal postural responses after vestibular stimulation (Smoot Reinert et al., 2015). Vestibular issues are likely under-reported in children with ASD and may go unrecognized. This is significant as unidentified, or untreated vestibular issues in childhood can have a number of poor outcomes. In particular, normal vestibular function is required for normal posture and gaze, development of fine motor skills, proper cognitive development and educational performance, and emotional and social behavior (reviewed in Van Hecke et al., 2019). The cerebellum also receives input from a number of non-motor/somatosensory sources and projects widely over the neuroaxis. Consistent with these projections, there is evidence that the cerebellum is involved in multiple functions beyond motor coordination via projections to the hippocampus, amygdala, and septal nuclei (Heath, 1973; Heath and Harper, 1974).

## Summary and Conclusions

The literature provides abundant evidence for both structural and functional hearing deficits in ASD. These findings are consistent with key signs and symptoms, specifically that individuals with ASD appear unaware when people talk to them, but respond to non-verbal sounds, repeat words or phrases in place of normal speech and have abnormal reactions to sensory stimulation (CDC.gov, 2021). Importantly, both functional and anatomical investigations indicate these auditory problems are present at birth. Consistent with these findings, we propose that qualitative ABRs or ASRs be used to screen for ASD risk (Lukose et al., 2013; Mansour and Kulesza, 2020; in accordance with other researchers: Grewe et al., 1994). Observations of spontaneous nystagmus and testing of VORs are also simple and non-invasive. The literature also provides evidence for vestibular dysfunction in children diagnosed with ASD. Similarly, if these vestibular issues can be identified in the early postnatal period, they could lead to early diagnosis of ASD and/or raise suspicion for other neurodevelopmental conditions. The addition of vestibular assessment to a neonatal auditory testing panel, we believe, would only improve the diagnostic power of early screening. Accordingly, future research in this area should be centered on determining the predictive value of combined auditory and vestibular testing on newborns for diagnosis of ASD and/or other neurodevelopmental disorders. Currently, almost all US states require newborn hearing screening^1^, although these tests are considered only on a pass/fail basis. We propose these initial screenings be evaluated on a quantitative basis to better stratify the risk of an ASD diagnosis. The addition of simple, noninvasive vestibular screening will only increase the value of auditory and vestibular assessment. Of course, the goal of early detection and diagnosis of ASD is early intervention to improve the quality of life and ensure the best possible outcomes and social/academic integration. There is evidence that early intervention for children with ASD focusing on eye contact, gesturing, and vocalizations results in significant improvements in the child’s language and social interactions (Wong and Kwan, 2010).

## Author Contributions

YM: literature review, made figures, and revised the manuscript. AB: literature review and revised manuscript. RK: literature review, wrote draft, made figures, and revised the manuscript. All authors contributed to the article and approved the submitted version.

## Conflict of Interest

The authors declare that the research was conducted in the absence of any commercial or financial relationships that could be construed as a potential conflict of interest.

## Publisher’s Note

All claims expressed in this article are solely those of the authors and do not necessarily represent those of their affiliated organizations, or those of the publisher, the editors and the reviewers. Any product that may be evaluated in this article, or claim that may be made by its manufacturer, is not guaranteed or endorsed by the publisher.

## Footnotes

^1^ CDC.gov/ncbddd/hearingloss/screening.html

## Abbreviations

A1: primary auditory cortex
ABR: auditory brainstem response
AN: auditory nerve
ASD: autism spectrum disorder
ASR: acoustic stapedial reflex
BIC: binaural interaction components
CNIC: central nucleus of the inferior colliculus
CN: cochlear nuclei
DCN: dorsal cochlear nucleus
DNLL: dorsal nucleus of the lateral lemniscus
DPOAE: distortion product otoacoustic emission
IC: inferior colliculus
INLL: intermediate nucleus of the lateral lemniscus
ITD: interaural timing difference
LL: lateral lemniscus
LOC: lateral olivocochlear
LSO: lateral superior olive
MOC: medial olivocochlear
MSO: medial superior olive
NT: neurotypical
OAE: otoacoustic emission
SOC: superior olivary complex
SPON: superior paraolivary nucleus
Tz: trapezoid body
VCN: ventral cochlear nucleus
vMG: ventral nucleus of the medial geniculate
VNLL: ventral nucleus of the lateral lemniscus
VNTB: ventral nucleus of the trapezoid body
VOR: vestibulo-ocular reflex.
